# Molecular Recognition in Glycolaldehyde, the Simplest Sugar: Two Isolated Hydrogen Bonds Win Over One Cooperative Pair

**DOI:** 10.1002/open.201200031

**Published:** 2012-10-12

**Authors:** Jonas Altnöder, Juhyon J Lee, Katharina E Otto, Martin A Suhm

**Affiliations:** a Institut für Physikalische Chemie, Universität GöttingenTammannstr. 6, 37077 Göttingen (Germany) E-mail: msuhm@gwdg.de

**Keywords:** ab initio calculations, carbohydrates, conformational analysis, infrared spectroscopy, molecular recognition, Raman spectroscopy

## Abstract

Carbohydrates are used in nature as molecular recognition tools. Understanding their conformational behavior upon aggregation helps in rationalizing the way in which cells and bacteria use sugars to communicate. Here, the simplest *α*-hydroxy carbonyl compound, glycolaldehyde, was used as a model system. It was shown to form compact polar *C*_2_-symmetric dimers with intermolecular O–H⋅⋅⋅O=C bonds, while sacrificing the corresponding intramolecular hydrogen bonds. Supersonic jet infrared (IR) and Raman spectra combined with high-level quantum chemical calculations provide a consistent picture for the preference over more typical hydrogen bond insertion and addition patterns. Experimental evidence for at least one metastable dimer is presented. A rotational spectroscopy investigation of these dimers is encouraged, also in view of astrophysical searches. The binding motif competition of aldehydic sugars might play a role in chirality recognition phenomena of more complex derivatives in the gas phase.

## Introduction

Carbohydrates are among the most powerful molecular recognition tools used in nature.^1^ Their delicate balance between intramolecular, intermolecular and solvent hydrogen bonds together with a restricted conformational flexibility is at the heart of their recognition power and deserves detailed investigation at all levels of molecular complexity.^2^, ^3^

*α*-Hydroxy carbonyl compounds model hydrogen bonding in the open form of reducing sugars. They have been shown to exhibit remarkable chirality recognition effects in the gas phase.^4^ In particular, the tetrameric complex of methyl lactate switches radically between different hydrogen-bond topologies when both enantiomers are present instead of just one.^5^ Binding between hydroxy groups can be competitive with isolated hydrogen bonds between a hydroxy group and a carbonyl group, depending on cooperative effects. Because there are geometric arrangements where both hydrogen-bond types can be realized at the same time, the binding situation is often quite complex.^6^ Additionally, aromatic contacts, for example, can tip the balance between intra- and intermolecular hydrogen-bond patterns rather easily.^7^ As a consequence, even dimeric interactions remain poorly understood in these model compounds.^6^, ^8^–^10^

To elucidate the subtle balance between intramolecular and two different kinds of intermolecular hydrogen bonding, it makes sense to study the simplest prototype,^11^ which has even been detected in interstellar space:^12^, ^13^ glycolaldehyde (CH(=O)CH_2_OH). Aldol reactions of glycolaldehyde catalyzed by dipeptides can lead to pentose sugars in an enantioselective way,^14^ adding to the importance of glycolaldehyde in prebiotic chemistry. In solid form, glycolaldehyde adopts a cyclic dimeric hemiacetal arrangement,^15^, ^16^ but in the gas phase, it provides the two essential functional groups for this study, a hydroxy group directly neighboring a carbonyl group, with which it can form a relatively strong internal hydrogen bond.^17^–^20^ At low temperature, other conformers can be safely neglected,^19^, ^20^ unless specifically prepared.^18^ Glycolaldehyde can dimerize via hydrogen bonds by inserting one hydroxy group into the internal hydrogen bond of the other, as seen in the water–glycolaldehyde complex.^21^ Alternatively, one monomer unit can add with its hydroxy group to the hydroxy group of the other, as seen for ethanolamines with aromatic alcohols.^4^ If both intramolecular hydrogen bonds survive, there is the option to form sandwich complexes between the two planar skeleton units, such as those observed in lactone dimers.^22^ Finally, the two monomers can also adopt chiral conformations by twisting the hydroxy group out of the symmetry plane. Two homoconfigured units can form a compact *C*_2_-symmetric dimer by realizing two intermolecular C=O⋅⋅⋅H–O bonds. The corresponding heteroconfigured *C*_i_-symmetric dimer is higher in energy and less compact. This is also true for other dimer structures that can be found on the potential energy hypersurface.

The present study aims at characterizing the most stable dimer structures of glycolaldehyde; larger clusters and metastable aggregates^23^ are beyond its scope.

## Results and Discussion

### Dimer potential energy hypersurface

**Figure 1:**
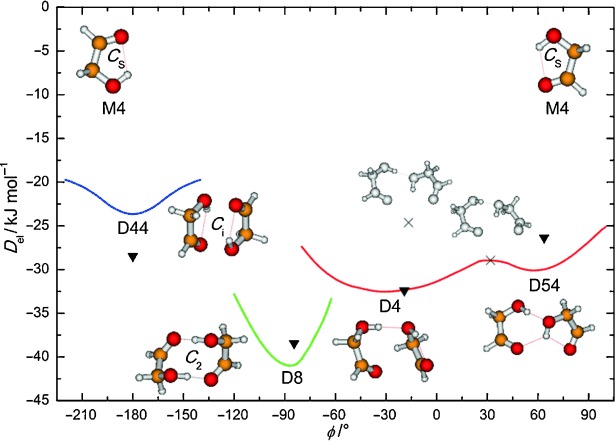
Important local minima (red: cooperative; green: isolated; blue: no intermolecular hydrogen bonds) and first-order saddles (×) on the glycolaldehyde dimer hypersurface along the O_1_–O_1_(H)–O_2_(H)–O_2_ dihedral angle (*ϕ*) at the B97D level. Electronic binding energies (*D*_el_) at the CCSD(T) extrapolated level are shown as triangles. Colored structures correspond to minima, grey-scale structures to saddle points.

We start with a simplified description of the complex inter-/intramolecular potential energy hypersurface of glycolaldehyde dimer (Figure 1). When two internally hydrogen-bonded monomers meet, they can form sandwich-like dimers (D) without significant barrier. These are abbreviated as D44 to illustrate the two hydrogen-bonded rings of four heavy atoms conserved from the monomers (M), which are denoted M4. The sandwich can be conrotatory (*C*_i_ symmetry) and is then relatively stable with respect to the formation of intermolecular hydrogen bonds. The disrotatory variant (*C*_2_ symmetry) is more amenable to ring opening and can cascade down to an interesting D4 docking structure, in which one monomer is conserved while the other hydroxy group docks to it via an OH⋅⋅⋅OH hydrogen bond. An important driving force for this is the cooperative OH_1_⋅⋅⋅OH_2_⋅⋅⋅O_2_=C pattern, as well as the persistent compactness. A more classical insertion of OH_1_ into the second monomer, which leads to a D54 topology, is less favorable but closely connected. A reason for its inferior stability might be the decreased compactness; it might also be due to the competitive rather than cooperative nature of the binding of the two carbonyl groups to one of the hydroxy groups. The partially cooperative D4 and D54 topologies are separated by relatively broad barriers from the global minimum structure D8, which features two isolated OH⋅⋅⋅O=C hydrogen bonds in a *C*_2_-symmetric flexible ring arrangement. While these insights have been obtained from extensive relaxed scans on the B97D hypersurface along the global dihedral coordinate (*ϕ*) spanned by the four oxygen atoms involved and for other internal coordinate choices, they are qualitatively preserved at higher levels, as indicated in Figure 1 and Table 1. The strong hydrogen bonds present in the key structures are quantified in Table 2. It would be highly interesting to map out the network of interconnected transition states and minima in a systematic way,^24^ but the present analysis is sufficient to set the scene for the supersonic jet experiment in the topology-sensitive O–H stretching region.

**Table 1:** Calculated electronic (Δ*E*_el_) and zero-point corrected (Δ*E*_0_) energies of D4, D44 and D54 relative to structure D8 (see Figure 1)[a]

Structure[b]	Method[c]	D4		D44 (*C*_i_)		D54	
		Δ*E*_el_	Δ*E*_0_	Δ*E*_el_	Δ*E*_0_	Δ*E*_el_	Δ*E*_0_
B3LYP	B3LYP	2.87	2.70	(20.79)	Saddle	4.01	3.13
B3LYP-D3	B3LYP-D3	8.85	8.77	16.55	15.86	12.89	11.23
B97D	B97D	8.43	7.87	17.30	15.68	10.86	9.76
MP2	MP2	0.07	2.21	10.93	11.48	5.95	6.74
MP2/aug	MP2/aug	4.87	4.78	9.33	8.67	10.31	8.67
MP2/aug	CCSD(T)[d]	6.05	5.97	9.96	9.30	12.09	10.44

[a] Δ*E* values are given in kJ mol^−1^.

[b] Level of structure optimization.

[c] Energy calculation method.

[d] MP2/aug with higher order CCSD(T) corrections.

**Table 2:** Inter- and intramolecular (O–)H⋅⋅⋅O distances (*d*_H⋅⋅⋅O_) in dimer structures optimized at the MP2/aug level—the highest level at which structural optimization was carried out)[a]

*d*_H⋅⋅⋅O_ [pm]	D8 (*C*_2_)	D4	D44 (*C*_i_)	D54
O–H⋅⋅⋅O=C (intramol)	–	202	208	211
O–H⋅⋅⋅O=C (intermol)	196	–	–	216
O–H⋅⋅⋅O–H (intermol)	–	182	–	181

[a] Only distances shorter than 220 pm are included; the M4 monomer (O–)H⋅⋅⋅O(=C) distance is 208 pm. Note the cooperative strengthening of the (O–)H⋅⋅⋅O(=C) bond in D4 relative to M4. B3LYP-D3 values are usually within 5 %; B97D deviations are larger for intramolecular contacts.

### IR and Raman jet spectra

**Figure 2:**
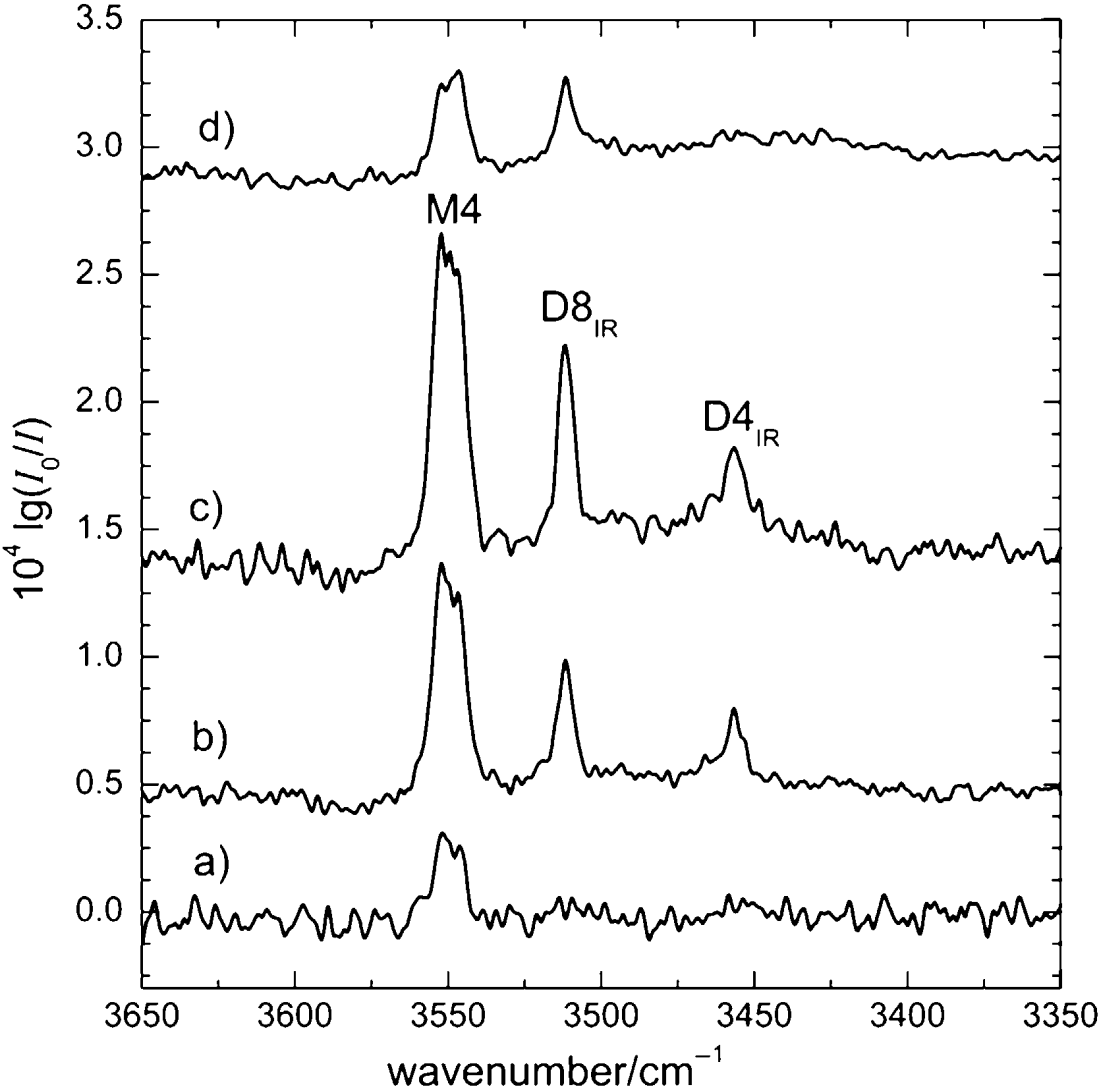
IR jet spectra of glycolaldehyde at varying concentrations and carrier gases; a–c) carrier gas 1.5 bar He, oven temperature (*θ*)=70, 80 and 90 °C, respectively; d) carrier gas 1.0–1.5 bar Ar, *θ*=70 °C.

Figure 2 shows infrared (IR) supersonic jet spectra as a function of glycolaldehyde concentration. At low concentrations (trace a)), only the M4 monomer peak at 3549 cm^−1^ is visible. With increasing concentration in the He carrier gas, two red-shifted peaks emerge at 3512 (D8_IR_) and 3457 cm^−1^ (D4_IR_). Their similar, although not identical, scaling with concentration suggests that they are both due to dimers and that D8_IR_ belongs to the more stable dimer, as it profits from an increasing number of intermolecular collisions. This is confirmed in the upper trace, where the carrier gas has been switched to Ar. Now, the D4_IR_ band has vanished due to the ten-times-larger mass of the colliding rare gas atoms, which improves the relaxation to the global minimum structure.

**Figure 3:**
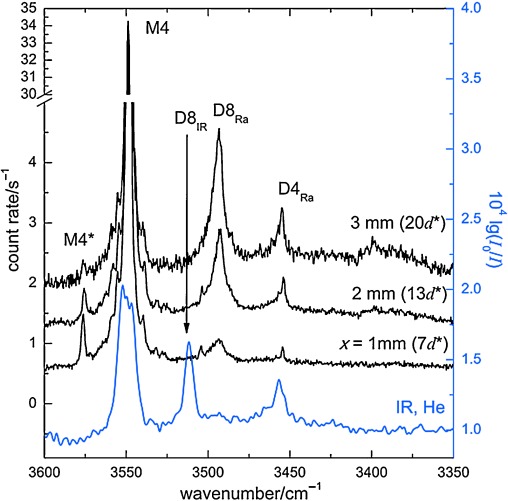
Raman jet spectra recorded at different nozzle distances (*x*) scaled to equal monomer band intensity. Reduced nozzle distances are given in parentheses where d*=0.15 mm is the width of the slit, the oven temperature was 85 °C. An average of the IR spectra b) and c) from Figure 2 is also included for comparison (blue).

Figure 3 shows Raman spectra scaled to equal monomer intensity recorded at different distances from the nozzle. Red-shifted to the dominant monomer band, there are three bands at 3504, 3494 (D8_Ra_), and 3455 cm^−1^ (D4_Ra_), and a single blue-shifted band is centered at 3576 cm^−1^ (M4*). In the upper trace, there is an additional broad band around 3399 cm^−1^ that stems from trimers or higher clusters and will not be discussed further. The bands differ in their dependence on the nozzle distance: The M4* band decreases with larger distances, the band at 3504 cm^−1^ remains more or less unchanged, and the other two increase. The D4_Ra_ band is clearly the counterpart of D4_IR_, with almost identical band position within calibration and temperature variations. The dominant IR dimer band D8_IR_ has its Raman counterpart D8_Ra_ at 3494 cm^−1^. The splitting (Δ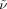) of 18 cm^−1^ supports two equivalent local OH oscillators, coupled through space or along the bonds into an antisymmetric (very IR active) and a symmetric (more Raman active) combination. Because the band at 3504 cm^−1^ has a scaled intensity rather independent of the distance of the nozzle, it is most probably a monomer band. The intensity of this band of less than 1 % relative to the dominant monomer hydroxy band leaves room for many interpretations including a C=O overtone (although that would be expected to appear at a wavenumber approximately 10–20 cm^−1^ lower than that observed) and other combinations, possibly enhanced by Fermi resonance. ^13^C isotope effects can be excluded since calculated harmonic wavenumbers (B3LYP and B97D) of ^13^C-substituted monomers shift the O–H stretching wavenumber by less than 0.1 cm^−1^. The distance-sensitive, blue-shifted band at 3576 cm^−1^ (M4*) is most likely a monomer hot band. Alternatively, it belongs to a metastable dimer that is converted to a more stable one during the expansion.

**Figure 4:**
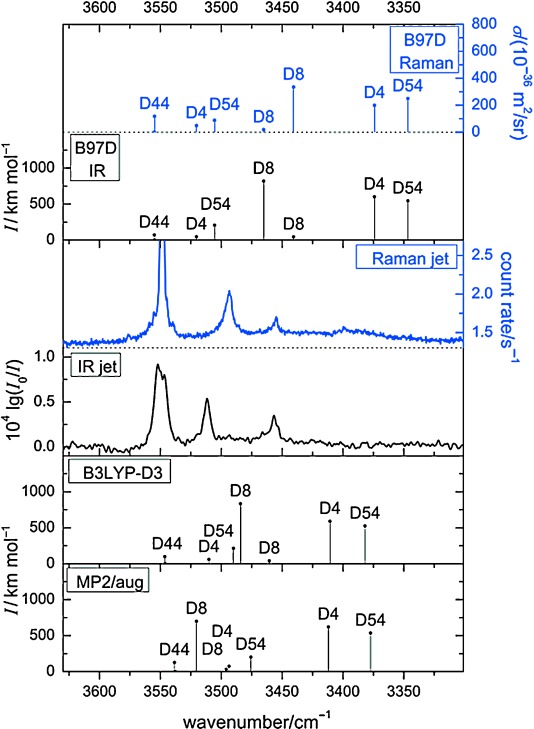
Harmonic O–H stretching wavenumbers (*ω*), IR band strengths (*I*), and Raman scattering cross sections (*σ*^Ra^) of dimers D8, D4, D44 and D54 calculated at the B97D, B3LYP-D3, and MP2/aug levels. All wavenumbers are shifted by the difference between the calculated and experimental monomer wavenumber at each level; experimental IR (black) and Raman (blue) spectra are also included for comparison. The D8 and D4 fractions in the expansion are estimated at ≈5 %, thus explaining the low abundance of larger clusters.

Spectral predictions assist the assignment, although quantitative shifts are difficult to obtain for this subtle balance between intra- and intermolecular hydrogen bonds. Figure 4 shows such spectral predictions for the dimers after shifting the predicted harmonic wavenumbers such that M4 coincides with the experimental value. The purpose of the shift is to correct in first order for the O–H stretching anharmonicity shift of about 160 cm^−1^[25] and for other deficiencies. The magnitude of the shift (−85 cm^−1^ for B97D; −167 for MP2/aug; −148 for B3LYP-D3) shows that B97D predicts an OH oscillator that is too soft.

We note that the harmonic B3LYP-D3 M4 IR intensity (57 km mol^−1^) compares well to an anharmonic calculation^26^ (54 km mol^−1^) and allows to estimate the average monomer density at ≈10^14^ cm^−3^ for trace a) in Figure 2. The full width at half maximum of the monomer O–H stretching fundamental is also of interest. In the FTIR spectra, it is dominated by residual rotational structure, but in the Raman spectra, it amounts to 2 cm^−1^—close to the instrument resolution due to Q-branch dominance. This is clearly less than the recent observation in a *p*-H_2_ matrix^27^ supporting the presence of inhomogeneous contributions in the quantum matrix. The matrix shift of −11 cm^−1^ also indicates a substantial interaction of the glycolaldehyde molecules with the *p*-H_2_ matrix.

Assuming equal populations of D8, D4, D45 and D44, it is clear that D44 would be least visible in the IR spectra. The sign of its predicted shift from the monomer is inconsistent but in all cases the shift is rather small. It cannot be ruled out that the blue-shifted band (M4*) or any of the weak bands on the monomer slope observed in the Raman spectra is caused by this dimer. The other three isomers have similar IR visibility, but D54 can be ruled out because of its consistently large shift and metastability with respect to conversion to D4. This metastability increases from 2.5 kJ mol^−1^ at the B97D level to 6 kJ mol^−1^ at the CCSD(T)-corrected level. It decreases to about 2 kJ mol^−1^ (0.3 kJ mol^−1^) when including vibrational zero-point energy, thermal excitation and entropy contributions in the rigid rotor and harmonic oscillator approximation on the B3LYP-D3 level at 100 K (300 K). D4 itself is a likely candidate for the most red-shifted peak that does not survive Ar relaxation. The relative intensity pattern of D8_IR_ and D8_Ra_, as well as the relative position of the peaks between the monomer and D4, makes their assignment to the D8 structure likely. In addition, the experimentally observed splitting (Δ
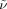
=18 cm^−1^) matches the predicted Davydov splitting quite well (Table 3).

**Table 3:** Calculated harmonic wavenumbers (*ω*), IR band strengths (*I*^IR^), and Raman scattering cross sections (*σ*^Ra^) of the O–H stretching vibrations in glycolaldehyde monomer (M) and dimers (D)

		B97D			MP2/aug		B3LYP-D3	
	*ω* [cm^−1^]	*I*^IR^ [km mol^−1^]	*σ*^Ra^ [10^−36^ m^2^ sr^−1^]	*ω* [cm^−1^]	*I*^IR^ [km mol^−1^]	*μ*[a] [D]	*ω* [cm^−1^]	*I*^IR^ [km mol^−1^]
M4	3634	42	64	3716	75	2.97	3697	57
D8	3550	820	19	3688	701	3.61	3632	835
	3525	45	335	3663	34	–	3608	41
D4	3605	47	49	3661	75	1.03	3658	61
	3459	601	199	3579	622	–	3558	591
D44	3640	72	0	3706	128	0	3694	100
	3640	0	118	3705	0	–	3694	0
D54	3590	206	88	3643	203	1.62	3638	215
	3431	546	250	3544	538	–	3530	526

[a] Dipole moments (*μ*) indicate that the most stable dimer should have a high microwave visibility. The calculated monomer value compares reasonably well to the experimental value of 2.34(1) D.^11^

Based on this assignment, important conclusions can be drawn about the performance of quantum chemical methods for the frequency shift between an internally hydrogen-bonded hydroxy group and an intermolecularly bonded hydroxy group. Although the B97D approach captures the energetics reasonably well (Table 1), it strongly overestimates the red shift of the D8 and D4 structures. This is in line with the underestimation of the O–H stretching wavenumber and a well-known deficiency of pure density functionals. The B3LYP-D3 and MP2/aug predictions bracket the experimental values for the D8 O–H stretching fundamentals, and the deviations are well within the expected error due to the neglect of anharmonicity. For the intense D4 O–H stretching transition, the overestimate of the red shift is sizeable. Part of it is due to weakening of the OH⋅⋅⋅OH hydrogen bond by librational zero-point motion of the donor. Its harmonic wavenumber increases by more than 60 % compared with the monomer (MP2/aug, B3LYP-D3). The resulting increase in the OH/librational coupling constant (*x*_OH,lib_) will be on the order of 20 cm^−1^ (see below), half of which must be added to the harmonic O–H stretching prediction.

Even for the monomer, such libration-stretching couplings are important because they can give rise to blue-shifted hot bands. For this purpose, Table 4 contains anharmonic predictions^28^ for the three lowest monomer vibrations and compares them with previous anharmonic calculations^19^, ^27^, ^29^ and experimental estimates.^11^, ^30^ The hydrogen bonded ring puckering mode *ν*_18_ (A“) affects the O–H stretching mode somewhat. The hydrogen-bond breathing mode *ν*_12_ (A′) has less effect on *ν*_OH_. Finally, *ν*_17_ (A”) near 300 cm^−1^ is closest to hydrogen-bond libration, and indeed, it has a nearly 10 % positive anharmonicity contribution *x*_OH,17_. Therefore, the hot transition from *ν*_17_ to *ν*_17_+*ν*_OH_ is predicted to be shifted by ≈30 cm^−1^ from the *ν*_OH_ fundamental. This provides support for the monomer hot band assignment of the M4* band at 3576 cm^−1^, which is shifted by 27 cm^−1^ from the O–H fundamental. The evaluation of the relative band heights in Raman spectra recorded at distances of 1, 2 and 3 mm (Figure 3) together with the 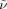
_17_ value of ≈300 cm^−1^ leads to reasonable vibrational temperatures of about 120, 106, and 93 K, respectively, assuming unchanged Raman scattering cross sections.

**Table 4:** Predicted anharmonic wavenumbers (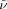
_i_) and nondiagonal anharmonicity constants (*x*_OH,i_) for the three lowest frequency vibrations. Experimental data are also included for comparison[a]

Method	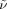 _18_ (A“)	*x*_OH,18_	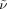 _12_ (A′)	*x*_OH,12_	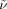 _17_ (A”)	*x*_OH,17_
B3LYP/6-311+G^*^	187	10	266	4	304	21
B3LYP/6-311++G^*^^*^	198	11	266	2	319	29
B3LYP/6-311++G^*^^*^^29^	187	11	268	2	298	35
B3LYP/TZVP	183	12	267	2	294	29
MP2/6-311++G(d,p)^27^	(290)	–	280	–	307	–
MP2/cc-pVTZ	210	8	280	2	359	34
MP2/cc-pVTZ (2D Fourier fit)^19^	199	–	–	–	369	–
MW estimate^30^	≈195	–	≈260	–	≈313	–
MW estimate^11^	195±30	–	260±40	–	–	–
This work ( 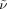 _M4^*^_− 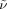 _M4_)	–	–	–	–	–	27

[a] Values for 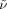
_i_ and *x*_OH,i_ are given in reciprocal centimeters (cm^−1^).

**Figure 5:**
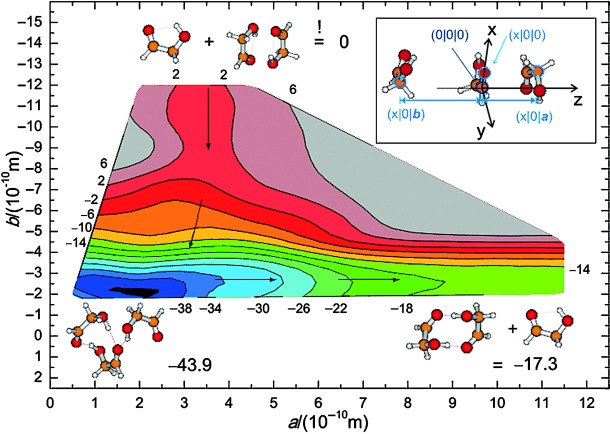
Representative relaxed scan of the 66-dimensional potential energy hypersurface of B97D glycolaldehyde trimer along two intermolecular separation coordinates *a* and *b*, keeping atoms circled in light blue in the *x*,*z* plane and the central carbonyl carbon atom circled in dark blue in the origin of the coordinate system. A seemingly barrierless exothermic path from a monomer M4 approaching a dimer D44 (upper left) to dimer D8 expelling M4 (lower right) via a trimer (lower left) is marked with arrows. Numbers indicate energies in kJ mol^−1^ relative to the sum of non-interacting M4 and D44 structures.

The experimental spectra are consistent with a trapping of D4 in the expansion despite an energy of ≈6 kJ mol^−1^ above the D8 isomer (Table 1). This must be related to relatively complex pathways from D4 to D8 combined with efficient formation of D4 upon monomer collision. Indeed, a disrotatory sandwich complex of two glycolaldehyde units can be imagined to transform rather smoothly into the D4 structure. This is not the case for the conrotatory *C*_i_-symmetric sandwich complex D44, for which the spectral features might be hidden under the monomer hydroxy peak. As illustrated in Figure 1, it is relatively stable with respect to isomerization. However, this is no more true when a third glycolaldehyde unit approaches the D44 structure. As illustrated in Figure 5, this can lead to the nearly barrierless formation of a transient trimer, which then expels another monomer on its way to a D8 dimer. Such an isomerization mechanism was recently postulated for the imidazole–water complex, which is formed quantitatively in its most stable isomer in a supersonic jet expansion,^31^ despite an undeniable trapping potential for the metastable isomer with opposite donor/acceptor roles.^32^ A similar process involving an Ar atom might be responsible for the depletion of D4 in favor of D8.^33^ Ar attachment to D4 leads to sufficient internal energy for isomerization to D8, and when the complex realizes this structure, there is enough energy available to expel the Ar atom. While still speculative, such mechanisms could explain the elusiveness of the D44 and D54 structures in the expansion as well as the conversion of D4 into D8 with a suitable collision partner. By varying the strength of the intramolecular hydrogen bond, one should be able to stabilize sufficient amounts of “nonreactive” sandwich complexes of D44 type or to conserve intramolecular hydrogen bonds by simply docking onto the acceptor sites of the cyclic monomer. Glycolaldehyde seems to offer just the right balance to tip the kinetically favored docking complex over into the global minimum structure, when the collision partner is switched from He to Ar or the number of collisions is increased. Rather frequently, this process remains inactive.^4^

### Comparison to related compounds

Glycolaldehyde has been shown to be a system where suitable supersonic expansion leads to an essentially monoisomeric composition of the dimer. We note that the closely related case of hydroxyacetone is much less straightforward.^6^ The extra methyl group in the acetone backbone lowers both the sandwich and docking dimers, because these structures can profit more from dispersion interactions. In the *C*_2_-symmetric D8 structure, the methyl groups stay relatively far apart. In this context, it is instructive to compare the dispersion energy contributions at the B3LYP-D3 structures for glycolaldehyde and hydroxyacetone dimers. B3LYP-D3 is the most reliable level at which the dispersion correction has been quantified. Relative to two monomers (in their most stable geometry), the gain is similar for the D8 structures (18 kJ mol^−1^ for glycolaldehyde; 20 kJ mol^−1^ for hydroxyacetone). For the D4 structure, it increases from 15 kJ mol^−1^ for glycolaldehyde to 26 kJ mol^−1^ for hydroxyacetone. Therefore, the isomer distribution in jet expansions of hydroxyacetone dimers remains more diverse than that of glycolaldehyde. However, the two systems are so closely related that the complete assignment of the glycolaldehyde case can assist and confirm the assignment of hydroxyacetone dimers, also validating theoretical descriptions. Experimentally, the monomeric O–H stretching fundamental is shifted by −24 cm^−1^ (B3LYP-D3 predicts −26 cm^−1^). Upon D8 dimerization, it shifts by 46–48 cm^−1^ to lower wavenumber for both systems, when the average between the IR- and Raman-prominent signal is taken (B3LYP-D3: 71–77 cm^−1^). As discussed before, the exaggeration of the harmonic red shift is in part due to neglect of the coupling between O–H stretching and librational motion. Not surprisingly, the size of the Davydov splitting is also exaggerated by theory (experiment: 13–18 cm^−1^; B3LYP-D3: 24–26 cm^−1^).^6^ The experimental decrease of the Davydov splitting upon methylation is not confirmed by calculation. This could hint at an anharmonic resonance, but other subtle effects are conceivable. Agreement for the D4 structure is also systematic. For glycolaldehyde, the intense O–H stretching band is red-shifted by 94 cm^−1^ (B3LYP-D3: 139 cm^−1^), whereas it is only shifted by ≈45 cm^−1^ in hydroxyacetone (B3LYP-D3: 62 cm^−1^).

The vapor pressure of glycolaldehyde is two orders of magnitude smaller than that of hydroxyacetone.^34^ One might suspect that most of this is due to chemical dimerization of the former, but it is claimed that monomeric glycolaldehyde was present in the condensed phase.^34^ This should be verified by vibrational spectra.

The subtle, dispersion-mediated interplay between isolated O–H⋅⋅⋅O=C hydrogen bonds and cooperative O–H⋅⋅⋅O–H⋅⋅⋅O arrangements reappears in the tetramer of chiral methyl lactate.^5^ Alternating *R*,*S*,*R*,*S* tetramers are able to optimize the methyl group interactions in a four-fold O–H⋅⋅⋅O=C coordinated, compact structure. Exchange of one or more of the units by its enantiomer causes a switch in the preferred structure to a cooperative O–H⋅⋅⋅O–H⋅⋅⋅O hydrogen bond pattern. Quantum chemical methods attempting to reproduce this configurational selectivity can be tested using the simpler glycolaldehyde and hydroxyacetone dimers. As in the case of methyl glycolate,^9^ removal of a methyl group stabilizes the isolated O–H⋅⋅⋅O=C pattern.

## Conclusion

Collisions between jet-cooled glycolaldehyde molecules near 100 K lead to the most stable dimer with isolated hydrogen bonds with a best estimate for the dissociation energy of 33 kJ mol^−1^ at 0 K, although other structures can be stabilized under suitable expansion conditions. We suggest a rotational spectroscopic study of the most stable polar (thus microwave-active) dimer, which might even exist in small quantities in the interstellar space and is probably thermodynamically more stable than the chemically bound dimer (according to B3LYP-D3 including harmonic zero-point vibrational energy).

The case of glycolaldehyde illustrates that cooperative hydrogen-bond patterns can be superseded by isolated bonds if the geometric constraints are favorable for the latter. This insight was obtained from a quantum chemically supported analysis of the O–H stretching range, whereas the C=O stretching region is complicated by Fermi resonance^23^ and weak dimer signals. Mere extension of the carbon backbone by one methylene unit outside the functional groups, as in hydroxyacetone, can completely change the isomer sequence due to dispersive interactions. This could provide a key to the versatile recognition features of carbohydrates. It will be interesting to see how the preferred binding motif of glycolaldehyde dimers survives in the *α*-hydroxy ester case. Larger clusters of these compounds lead to impressive and structurally resolved molecular recognition,^5^, ^9^ whereas the dimer spectra still remain to be fully understood.

## Experimental Section

**Quantum chemical calculations**: The potential energy hypersurface was explored at the B97D/TZVP/TZVPfit level (in brief B97D) and refined at the MP2/aug-cc-pVTZ level (MP2/aug), including zero-point energy corrections (*▵*ZPE) at the same level and higher order correlation corrections at the ▵CCSD(T)/6-311+G* level (CCSD(T)).^35^ For comparison, MP2/6-311+G* (MP2), B3LYP/6-311+G* (B3LYP), and dispersion corrected^36^ B3LYP-D3/def2-TZVP (B3LYP-D3) results are also reported. B3LYP-D3 calculations were carried out with Turbomole (V6.3.1),^37^ B97D with the Gaussian 09 program package,^38^ and all others with Gaussian 03.^39^ Differential Raman cross sections (σ^Ra^) were calculated at a 90 ° angle for *λ*=532 nm assuming a vibrational temperature of 100 K.

**Jet Fourier-transform infrared (FTIR) spectroscopy**: Spectra were recorded using a heatable double-slit nozzle (slit size=0.5×10 mm^2^ each) probed at ≈4 mm downstream. Descriptions of the apparatus can be found elsewhere.^7^ Glycolaldehyde, in the form of the chemically bound nonvolatile dimer, was purchased from Sigma–Aldrich and used without further purification. Glycolaldehyde was deposited on molecular sieve (3 Å) and heated in a glass tube between two poppet valves at 70–90 °C. Pulses of either He or Ar carrier gas with a duration of 0.3 s were guided through the glass tube, picking up small amounts (∼0.1 %) of monomeric glycolaldehyde. The mixture was expanded into an evacuated buffer volume, and the gas pulses were synchronized to FTIR scans (Bruker IFS 66v/S) using an InSb detector equipped with an appropriate optical bandpass filter. Typical spectra were obtained by co-adding 300–500 FTIR scans collected in 150–250 gas pulses.

**Raman spectroscopy**: Spontaneous Raman scattering was detected using a previously described setup.^40^ He (0.7 bar) was guided through a heatable saturator containing glycolaldehyde, and the gas mixture was expanded continuously through a slit nozzle (slit size=0.15×4.0 mm^2^). A Coherent Verdi V18 frequency-doubled Nd:YVO_4_ laser beam (cw, 18 W, *λ*=532 nm) was focused onto the expansion at different distances from the nozzle to reveal the spatial evolution of the expansion. Scattered light was collected perpendicular to jet and laser directions and focused on the entrance slit of a 1 m monochromator (McPherson model 2051 *f*/8.6) after filtering out the Rayleigh scattered photons by an edge filter. The dispersed photons were detected with a CCD camera (PI Acton, Spec-10: 400 B/LN, 1340×400 pixels) cooled with liquid N_2_. Ne I emission lines were used for wavelength calibration. Cosmic ray signals were removed by the comparison of block-averaged spectra.

Although traces of water are occasionally observed in the spectra, the spectral features discussed here do not correlate with concentration variations of this impurity. Our jet spectra show no evidence of the chemically bound dimer of glycolaldehyde.^41^
